# 2-(4-Chloro­phen­oxy)-*N*′-[2-(4-chloro­phen­oxy)acet­yl]acetohydrazide monohydrate

**DOI:** 10.1107/S160053681004050X

**Published:** 2010-10-20

**Authors:** Ting Chen, Xiaosong Tan

**Affiliations:** a Faculty of Material Science and Chemical Engineering, China University of Geosciences, Wuhan 430074, People’s Republic of China; b Key Laboratory of Pesticide & Chemical Biology, Ministry of Education, College of Chemistry, Central China Normal University, Wuhan 430079, People’s Republic of China

## Abstract

In the title compound, C_16_H_14_Cl_2_N_2_O_4_·H_2_O, the hydrazine and water mol­ecules are both located on twofold axes. The C—N—N—C torsion angle is −72.66 (1)° and the dihedral angle between the two benzene rings is 67.33 (1)°. In the crystal, mol­ecules are linked into a layer structure by a combination of O—H⋯O, N—H⋯O and C—H⋯O hydrogen bonds. Adjacent layers are linked into a three-dimensional network by Cl⋯Cl inter­actions [3.400 (2) Å]. C—H⋯π inter­actions are also observed.

## Related literature

For the synthesis and biological activity of title compound and its derivatives, see: Dovlatvan (1961 ▶). For the synthesis and biological activity of diacyl­hydrazine derivatives, see: Jia (2008 ▶); Zhang *et al.* (2005 ▶); Zhao *et al.* (2008 ▶). For a related structure, see: Jiang *et al.* (2009 ▶).
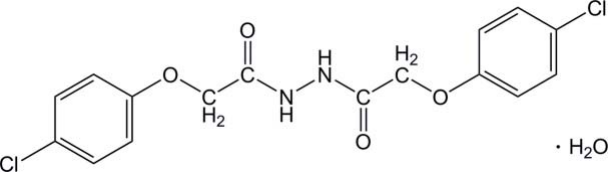


## Experimental

### 

#### Crystal data

C_16_H_14_Cl_2_N_2_O_4_·H_2_O
*M* *_r_* = 387.21
Monoclinic, 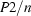

*a* = 4.8462 (9) Å
*b* = 5.4411 (10) Å
*c* = 33.521 (6) Å
β = 90.840 (3)°
*V* = 883.8 (3) Å^3^
*Z* = 2
Mo *K*α radiation
μ = 0.40 mm^−1^
*T* = 292 K
0.10 × 0.04 × 0.02 mm

#### Data collection

Bruker SMART CCD area-detector diffractometer
9670 measured reflections
2013 independent reflections
1380 reflections with *I* > 2σ(*I*)
*R*_int_ = 0.059

#### Refinement

*R*[*F* ^2^ > 2σ(*F* ^2^)] = 0.056
*wR*(*F* ^2^) = 0.169
*S* = 1.06
2013 reflections
121 parameters
2 restraints
H atoms treated by a mixture of independent and constrained refinement
Δρ_max_ = 0.34 e Å^−3^
Δρ_min_ = −0.27 e Å^−3^

### 

Data collection: *SMART* (Bruker, 2001 ▶); cell refinement: *SAINT* (Bruker, 2001 ▶); data reduction: *SAINT*; program(s) used to solve structure: *SHELXS97* (Sheldrick, 2008 ▶); program(s) used to refine structure: *SHELXL97* (Sheldrick, 2008 ▶); molecular graphics: *SHELXTL* (Sheldrick, 2008 ▶); software used to prepare material for publication: *SHELXTL*.

## Supplementary Material

Crystal structure: contains datablocks I, global. DOI: 10.1107/S160053681004050X/vm2047sup1.cif

Structure factors: contains datablocks I. DOI: 10.1107/S160053681004050X/vm2047Isup2.hkl

Additional supplementary materials:  crystallographic information; 3D view; checkCIF report

## Figures and Tables

**Table 1:** Hydrogen-bond geometry (Å, °) *Cg*1 is the centroid of the C1–C6 ring.

*D*—H⋯*A*	*D*—H	H⋯*A*	*D*⋯*A*	*D*—H⋯*A*
C5—H5⋯O2^i^	0.93	2.47	3.382 (3)	166
O3—H3*A*⋯O2^i^	0.82 (1)	1.96 (1)	2.765 (2)	169 (4)
N1—H1⋯O3	0.86 (1)	2.12 (2)	2.911 (3)	153 (3)
N1—H1⋯O1	0.86 (1)	2.26 (3)	2.633 (2)	107 (2)
C7—H7⋯*Cg*1^ii^	0.97	2.76	3.592 (1)	144

Symmetry codes: (i) 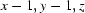; (ii) 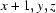.

**Fig. 1.**
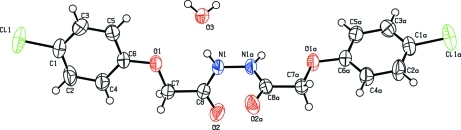
The molecular structure of the title compound, showing the atom-labeling scheme for the non-H atoms and 50% probability displacement ellipsoids.

**Fig. 2.**
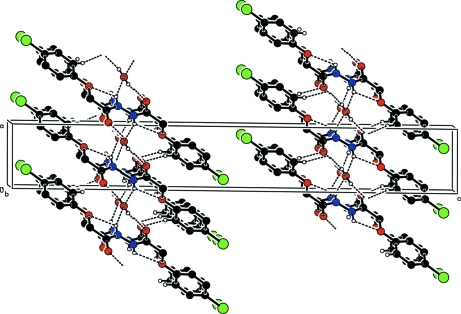
Two-dimensional layer structure by hydrogen bonding indicated as dashed lines.

**Fig. 3.**
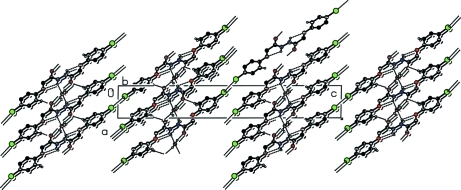
Three-dimensional network formed via Cl1···Cl1 (-x, -y, 1 - z) interactions.

## supplementary crystallographic information

### Comment

Most diacylhydrazine derivatives have insecticide activity (Zhang *et al.*, 2005; Jia, 2008; Zhao *et al.*, 2008). While in our research of herbicidal compounds, we found some diacylhydrazine derivatives showing herbicidal activity. We have synthesized the title compound and report its crystal structure here.

In the title compound (Fig. 1), the hydrazine and water molecules are both located on twofold axes. The torsion angle C8—N1—N1(-*x* + 5/2, *y*, -*z* + 1/2)—C8(-*x* + 5/2, *y*, -*z* + 1/2) is -72.66 (1)° and the dihedral angle between the two benzene rings is 67.33 (1)°. Intermolecular N—H···O and intramolecular O—H···O, C—H···O hydrogen bonds are found in the crystal structure (Table 1), and one C—H···π interaction [C7···*Cg*1(*x* + 1, *y*, *z*) = 3.592 (1) Å, *Cg*1 is the centroid defined by benzene atoms C1—C6] is also observed.

In the crystal packing, the molecules are linked into a two-dimensional layer structure by a combination of O—H···O, N—H···O and C—H···O hydrogen bonds (Fig. 2). These adjacent layers are linked into a three-dimensional network by the Cl1···Cl1(-*x*, -*y*, 1 - *z*) interaction (3.400 (2) Å, Fig. 3).

### Experimental

4-chlorophenoxyacetyl chloride (4.10 g, 20 mmol) was dissolved in toluene (20 ml), together with hydrazine hydrate (85%, 0.59 g, 10 mmol). The solution was stirred at room temperature and then pyridine (1.60 g, 20 mmol) was added dropwise. Then the solution was heated at 373 K for two hours. The product was isolated and recrystallized as a colorless solid from ethanol (yield 80.3%).

### Refinement

H atoms on C atoms were positioned geometrically and refined using a riding model with C—H = 0.93Å (aromatic) and 0.97Å (methylene). The *U*_iso_(H) values were set 1.2 times of their parent atoms. H atoms attached to N and O atoms were found from the difference maps and refined with restraints (N—H = 0.86 (1)Å and O—H = 0.82 (1) Å), and their thermal factors were set 1.2 times (for N) or 1.5 times (for O) of the parent atoms.

### Crystal data

**Table:**

C_16_H_14_Cl_2_N_2_O_4_·H_2_O	*F*(000) = 400
*M**_r_* = 387.21	*D*_x_ = 1.455 Mg m^−^^3^
Monoclinic, *P*2/*n*	Mo *K*α radiation, λ = 0.71073 Å
Hall symbol: -P 2yac	Cell parameters from 2333 reflections
*a* = 4.8462 (9) Å	θ = 3.7–26.5°
*b* = 5.4411 (10) Å	µ = 0.40 mm^−^^1^
*c* = 33.521 (6) Å	*T* = 292 K
β = 90.840 (3)°	Block, colourless
*V* = 883.8 (3) Å^3^	0.10 × 0.04 × 0.02 mm
*Z* = 2	

### Data collection

**Table:**

Bruker SMART CCD area-detector diffractometer	1380 reflections with *I* > 2σ(*I*)
Radiation source: fine-focus sealed tube	*R*_int_ = 0.059
graphite	θ_max_ = 27.5°, θ_min_ = 1.2°
phi and ω scans	*h* = −6→6
9670 measured reflections	*k* = −6→6
2013 independent reflections	*l* = −43→43

### Refinement

**Table:**

Refinement on *F*^2^	Primary atom site location: structure-invariant direct methods
Least-squares matrix: full	Secondary atom site location: difference Fourier map
*R*[*F*^2^ > 2σ(*F*^2^)] = 0.056	Hydrogen site location: inferred from neighbouring sites
*wR*(*F*^2^) = 0.169	H atoms treated by a mixture of independent and constrained refinement
*S* = 1.06	*w* = 1/[σ^2^(*F*_o_^2^) + (0.0947*P*)^2^] where *P* = (*F*_o_^2^ + 2*F*_c_^2^)/3
2013 reflections	(Δ/σ)_max_ < 0.001
121 parameters	Δρ_max_ = 0.34 e Å^−^^3^
2 restraints	Δρ_min_ = −0.27 e Å^−^^3^

### Special details

**Table:**

Geometry. All e.s.d.'s (except the e.s.d. in the dihedral angle between two l.s. planes) are estimated using the full covariance matrix. The cell e.s.d.'s are taken into account individually in the estimation of e.s.d.'s in distances, angles and torsion angles; correlations between e.s.d.'s in cell parameters are only used when they are defined by crystal symmetry. An approximate (isotropic) treatment of cell e.s.d.'s is used for estimating e.s.d.'s involving l.s. planes.
Refinement. Refinement of *F*^2^ against ALL reflections. The weighted *R*-factor *wR* and goodness of fit *S* are based on *F*^2^, conventional *R*-factors *R* are based on *F*, with *F* set to zero for negative *F*^2^. The threshold expression of *F*^2^ > σ(*F*^2^) is used only for calculating *R*-factors(gt) *etc*. and is not relevant to the choice of reflections for refinement. *R*-factors based on *F*^2^ are statistically about twice as large as those based on *F*, and *R*- factors based on ALL data will be even larger.

### Fractional atomic coordinates and isotropic or equivalent isotropic displacement parameters (Å^2^)

**Table:**

	*x*	*y*	*z*	*U*_iso_*/*U*_eq_	
C1	0.3940 (6)	0.2592 (5)	0.43058 (8)	0.0523 (7)	
C2	0.5608 (6)	0.4610 (6)	0.43082 (7)	0.0596 (8)	
H2	0.5626	0.5664	0.4527	0.072*	
C3	0.3829 (6)	0.1066 (5)	0.39794 (9)	0.0585 (7)	
H3	0.2647	−0.0281	0.3977	0.070*	
C4	0.7276 (5)	0.5091 (5)	0.39850 (7)	0.0504 (7)	
H4	0.8425	0.6460	0.3987	0.060*	
C5	0.5469 (5)	0.1529 (5)	0.36551 (8)	0.0499 (6)	
H5	0.5394	0.0499	0.3434	0.060*	
C6	0.7225 (5)	0.3534 (4)	0.36602 (6)	0.0389 (5)	
C7	1.0585 (5)	0.5868 (4)	0.33195 (7)	0.0412 (6)	
H7A	1.1768	0.5850	0.3556	0.049*	
H7B	0.9502	0.7367	0.3324	0.049*	
C8	1.2341 (5)	0.5841 (4)	0.29520 (6)	0.0391 (5)	
Cl1	0.1904 (2)	0.1932 (2)	0.47157 (2)	0.0884 (4)	
O1	0.8805 (3)	0.3820 (3)	0.33266 (4)	0.0459 (5)	
O2	1.4157 (4)	0.7389 (3)	0.29242 (6)	0.0582 (5)	
N1	1.1790 (4)	0.4132 (4)	0.26786 (5)	0.0394 (5)	
O3	0.7500	0.0536 (4)	0.2500	0.0484 (6)	
H1	1.042 (4)	0.316 (5)	0.2707 (9)	0.066 (9)*	
H3A	0.636 (6)	−0.036 (6)	0.2602 (11)	0.099*	

### Atomic displacement parameters (Å^2^)

**Table:**

	*U*^11^	*U*^22^	*U*^33^	*U*^12^	*U*^13^	*U*^23^
C1	0.0490 (15)	0.0682 (17)	0.0399 (14)	−0.0006 (12)	0.0127 (11)	0.0082 (12)
C2	0.0648 (18)	0.080 (2)	0.0345 (13)	−0.0098 (15)	0.0128 (12)	−0.0117 (13)
C3	0.0557 (16)	0.0530 (16)	0.0673 (18)	−0.0132 (12)	0.0187 (13)	0.0008 (13)
C4	0.0531 (15)	0.0583 (16)	0.0400 (13)	−0.0155 (12)	0.0092 (11)	−0.0082 (11)
C5	0.0516 (15)	0.0500 (14)	0.0485 (15)	−0.0076 (12)	0.0129 (11)	−0.0088 (11)
C6	0.0358 (12)	0.0482 (13)	0.0330 (12)	0.0004 (10)	0.0064 (9)	−0.0009 (9)
C7	0.0433 (13)	0.0442 (13)	0.0363 (12)	−0.0054 (10)	0.0079 (10)	−0.0026 (10)
C8	0.0381 (12)	0.0435 (13)	0.0357 (12)	−0.0001 (10)	0.0051 (9)	0.0044 (10)
Cl1	0.0842 (6)	0.1257 (8)	0.0562 (5)	−0.0160 (5)	0.0333 (4)	0.0175 (4)
O1	0.0480 (10)	0.0540 (10)	0.0362 (9)	−0.0121 (8)	0.0154 (7)	−0.0079 (7)
O2	0.0606 (12)	0.0641 (12)	0.0504 (11)	−0.0269 (9)	0.0149 (9)	−0.0052 (8)
N1	0.0383 (11)	0.0435 (11)	0.0368 (10)	−0.0052 (9)	0.0135 (8)	−0.0018 (8)
O3	0.0477 (15)	0.0424 (14)	0.0558 (15)	0.000	0.0216 (11)	0.000

### Geometric parameters (Å, °)

**Table:**

C1—C2	1.363 (4)	C6—O1	1.373 (2)
C1—C3	1.374 (4)	C7—O1	1.410 (3)
C1—Cl1	1.741 (2)	C7—C8	1.507 (3)
C2—C4	1.386 (3)	C7—H7A	0.9700
C2—H2	0.9300	C7—H7B	0.9700
C3—C5	1.379 (3)	C8—O2	1.223 (3)
C3—H3	0.9300	C8—N1	1.330 (3)
C4—C6	1.380 (3)	N1—N1^i^	1.390 (3)
C4—H4	0.9300	N1—H1	0.856 (10)
C5—C6	1.383 (3)	O3—H3A	0.815 (10)
C5—H5	0.9300		
			
C2—C1—C3	120.5 (2)	O1—C6—C5	115.4 (2)
C2—C1—Cl1	120.3 (2)	C4—C6—C5	119.9 (2)
C3—C1—Cl1	119.2 (2)	O1—C7—C8	111.02 (18)
C1—C2—C4	120.0 (2)	O1—C7—H7A	109.4
C1—C2—H2	120.0	C8—C7—H7A	109.4
C4—C2—H2	120.0	O1—C7—H7B	109.4
C1—C3—C5	120.1 (2)	C8—C7—H7B	109.4
C1—C3—H3	120.0	H7A—C7—H7B	108.0
C5—C3—H3	120.0	O2—C8—N1	124.5 (2)
C6—C4—C2	119.8 (2)	O2—C8—C7	118.1 (2)
C6—C4—H4	120.1	N1—C8—C7	117.39 (19)
C2—C4—H4	120.1	C6—O1—C7	116.86 (17)
C3—C5—C6	119.7 (2)	C8—N1—N1^i^	119.77 (17)
C3—C5—H5	120.1	C8—N1—H1	120 (2)
C6—C5—H5	120.1	N1^i^—N1—H1	119 (2)
O1—C6—C4	124.7 (2)		
			
C3—C1—C2—C4	−2.1 (4)	C3—C5—C6—C4	−1.7 (4)
Cl1—C1—C2—C4	178.4 (2)	O1—C7—C8—O2	−173.5 (2)
C2—C1—C3—C5	1.8 (4)	O1—C7—C8—N1	6.8 (3)
Cl1—C1—C3—C5	−178.6 (2)	C4—C6—O1—C7	−0.3 (3)
C1—C2—C4—C6	0.5 (4)	C5—C6—O1—C7	179.0 (2)
C1—C3—C5—C6	0.1 (4)	C8—C7—O1—C6	175.62 (18)
C2—C4—C6—O1	−179.4 (2)	O2—C8—N1—N1^i^	−4.2 (4)
C2—C4—C6—C5	1.4 (4)	C7—C8—N1—N1^i^	175.4 (2)
C3—C5—C6—O1	179.0 (2)		

Symmetry codes: (i) −*x*+5/2, *y*, −*z*+1/2.

### Hydrogen-bond geometry (Å, °)

**Table:**

*Cg*1 is the centroid of the C1–C6 ring.

**Table:**

*D*—H···*A*	*D*—H	H···*A*	*D*···*A*	*D*—H···*A*
C5—H5···O2^ii^	0.93	2.47	3.382 (3)	166
O3—H3A···O2^ii^	0.82 (1)	1.96 (1)	2.765 (2)	169 (4)
N1—H1···O3	0.86 (1)	2.12 (2)	2.911 (3)	153 (3)
N1—H1···O1	0.86 (1)	2.26 (3)	2.633 (2)	107 (2)
C7—H7···*Cg*1^iii^	0.97	2.76	3.592 (1)	144

Symmetry codes: (ii) *x*−1, *y*−1, *z*; (iii) *x*+1, *y*, *z*.
